# The application analysis of 8F ultrafine chest drainage tube for thoracoscopic lobectomy of lung cancer

**DOI:** 10.1186/s13019-021-01479-x

**Published:** 2021-04-21

**Authors:** Yongbin Song, Chong Zheng, Shaohui Zhou, Hongshang Cui, Jincong Wang, Jianxun Wang, Wenhao Wang, Lijun Liu, Junfeng Liu

**Affiliations:** 1 Graduate School, Hebei Medical University, 361 Zhongshan East Road, Shijiazhuang, 050051 Hebei Province People’s Republic of China; 2 Department of Thoracic Surgery, Hebei General Hospital, 348 West He-Ping Road, Shijiazhuang, 050051 Hebei Province People’s Republic of China; 3 Graduate School, Hebei North University, 11 Diamond South Road, High-tech Zone, Zhangjiakou, 075000 Hebei Province People’s Republic of China; 4 Ningbo University Medical School, 818 Fenghua Road, Jiangbei District, Ningbo, 315000 Zhejiang Province People’s Republic of China; 5 Department of Thoracic Surgery, Hebei Medical University Fourth Affiliated Hospital and Hebei Provincial Tumor Hospital, 12 Jiankang Road, Shijiazhuang, Hebei Province 050011 People’s Republic of China

**Keywords:** Lung cancer, Thoracoscopic lobectomy, Ultrafine chest drainage tube

## Abstract

**Background:**

Currently, thoracoscopic lobectomy is widely used in clinical practice, and postoperative placement of ultrafine drainage tube has advantages of reducing postoperative pain and accelerating postoperative recovery in patients. This study aimed to investigate the feasibility and safety of placement of 8F ultrafine chest drainage tube after thoracoscopic lobectomy and its superiority over traditional 24F chest drainage tube.

**Methods:**

A retrospective data analysis was conducted in 169 patients who underwent placement of 8F ultrafine chest drainage tube or 24F chest drainage tube with thoracoscopic lobectomy for lung cancer from January 2018 to December 2019. Propensity score matching (PSM) was used to reduce bias between the experimental group and the control group. After PSM, 134 patients (67 per group) were enrolled. The drainage time, the total drainage volume, postoperative hospital stay, postoperative pain score and postoperative complication of both groups were analyzed and compared.

**Results:**

Compared to group B, group A had lower pain scores on postoperative days 1, 2 and 3 (3.72 ± 0.65point vs 3.94 ± 0.67point, *P* = 0.027; 2.72 ± 0.93point vs 3.13 ± 1.04point, *P* = 0.016; and 1.87 ± 0.65point vs 2.39 ± 1.22point, *P* = 0.005), shorter drainage time (4.25 ± 1.79d vs 6.04 ± 1.96d, *P* = 0.000), fewer drainage volume (1100.42 ± 701.57 ml vs 1369.39 ± 624.25 ml, *P* = 0.021); and shorter postoperative hospital stay (8.46 ± 2.48d vs 9.37 ± 1.70d, *P* = 0.014). Postoperative complications such as subcutaneous emphysema, pulmonary infection, atelectasis, chest tube reinsertion and intrathoracic hemorrhage showed no differences between both groups (*P* > 0.05).

**Conclusion:**

Compared with 24F chest drainage tube, the application of an 8F ultrafine chest drainage tube after thoracoscopic lobectomy has significantly shortened the drainage time, reduced the total drainage volume, reduced the postoperative pain degree, shortened the hospital day, and effectively detected postoperative intrathoracic hemorrhage. So, it is considered as an effective, safe and reliable drainage method.

## Background

The surgical method for lung cancer has gradually changed from thoracotomy to minimally invasive operation, and currently thoracoscopic lobectomy is the most commonly adopted approach in clinical practice [1–4]. The placement of a thoracic drainage tube after lobectomy assists in mainly draining the blood and gas in the thoracic cavity, prevents reflux of exudate, reconstructs the normal negative pressure in the thoracic cavity, promotes lung expansion and prevents intrathoracic infection [5, 6]. However, postoperative chest drainage tube placement aggravates wound pain, lowering the efficiency of cough and inactivity of ambulating, as it is not conducive to early postoperative recovery [7–9]. The concept of enhanced recovery after surgery (ERAS) focused on optimizing perioperative measures, alleviating surgical stress, reducing complications and achieving the goal of accelerated recovery by combining with minimally invasive surgery [10]. Hence, in this study, the data of 77 patients who underwent placement of 8F ultrafine chest drainage tube after thoracoscopic lobectomy were retrospectively analyzed and compared with 92 patients who underwent traditional 24F chest drainage tube. The feasibility and safety of 8F ultrafine chest drainage tube placement after thoracoscopic lobectomy, and its advantages over 24F chest drainage tube were investigated.

## Methods

### Patient selection

The medical data of patients with lung cancer who underwent video-assisted thoracoscopic surgery (VATS) lobectomy and systematic mediastinal lymph node dissection (MLND) in the Department of Thoracic Surgery I, Hebei General Hospital between January 2018 and December 2019 were retrospectively analyzed. All the operations were performed by the same team of surgeons. The selection of which drainage tube was mainly based on the experiences of surgeons. The inclusion criteria were as follows: patients (1) who underwent preoperative examination or intraoperative frozen section confirmed non-small cell lung cancer (NSCLC); and (2) with clinical stage I to IIIA disease according to the 8th edition of the TNM classification of lung cancer [11]. The patients with the following conditions were excluded from this analysis: (1) preoperative complications of atelectasis, pulmonary infection or tuberculosis; (2) with hemothorax and empyema; (3) who had second lobectomy; (4) with extensive dense pleural adhesion; (5) with tumor invading the chest wall (T3, 6) with non-single lobectomy; and (7) with lung lobe and other thoracic organs that require simultaneous excision.

According to the above criteria, a total of 169 patients were enrolled. The patients had been classified for type of drainage treatment they received and accordingly were allocated into two groups: 77 patients with 8F ultrafine chest tube were allocated to the experimental group (group A) and 92 patients with 24F chest tube were allocated to the control group (group B). After propensity score matching, a total of 67 pairs of patients matched successfully. Before matching, the difference in postoperative pathological staging between the two groups was statistically significant. After matching, there was no statistically significant difference in baseline data between the two groups. The general data of the two groups were compared as shown in Table 1.

**Table 1:** Preoperative Characteristics of Patients and Tumors

Characteristics	Before PSM			After PSM		
	Group A (*n* = 77)	Group B (*n* = 92)	*P*-value	Group A (*n* = 67)	Group B (*n* = 67)	*P*-value
Gender (%)			0.552			0.596
Male	51.95	56.52		58.21	62.69	
Female	48.05	43.48		41.79	37.31	
Age (years)	59.35 ± 10.98	60.57 ± 9.13	0.577	59.60 ± 11.76	60.64 ± 9.35	0.570
Tumor location (%)			0.782			0.633
Upper right	33.77	30.43		37.31	35.82	
Middle right	6.49	9.78		5.97	5.97	
Lower right	20.78	15.22		23.88	14.93	
Upper left	19.48	22.83		22.39	26.87	
Lower left	19.48	21.74		10.45	16.42	
Postoperative pathological staging (%)			0.018			0.720
IA	77.92	55.43		74.63	68.66	
IB	3.90	17.39		4.48	5.97	
IIA	5.19	5.43		5.97	2.99	
IIB	5.19	7.61		5.97	7.46	
IIIA	7.79	14.13		8.96	14.93	
Pathological types (%)			0.619			0.360
Adenocarcinoma	68.83	65.22		70.15	62.69	
Squamous cell carcinoma	31.17	34.78		29.85	37.31	

Scale variables were expressed as median and range, and ordinal and nominal parameters as absolute numbers, and percent.

### Surgical approach

Patients in both groups underwent VATS lobectomy and systematic MLND, and single-lung ventilation was performed by placing them in lateral decubitus position. After disinfection, the observation hole was made in the 7th intercostal space of the midaxillary line. A 1.5 cm incision was made and a thoracoscope was inserted. For the lesion of the upper lobe, an incision (3 ~ 4 cm) was made in the 4th intercostal space of the anterior axillary line. For the lesion in the middle or lower lobe, an incision (3 ~ 4 cm) was made in the 5th intercostal space of the anterior axillary line. The ultrasonic scalpel and linear cut-close device were used without rib spreading to completely resect the blood vessels, bronchi, and lobes. For both groups A and B, the upper lobectomy was performed with double drainage tube, and single drainage tube was placed for cases undergoing middle or lower lobectomy.

**Fig. 1:**
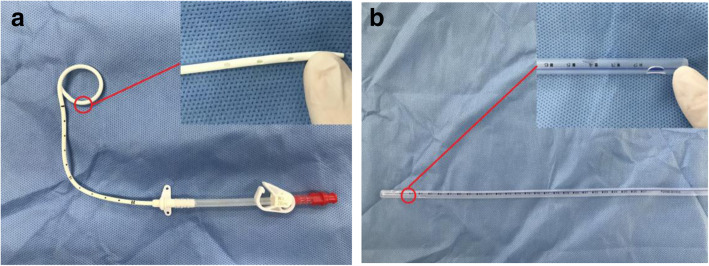
The appearance of chest drainage tube; **a** 8F chest drainage tube; **b** 24F chest drainage tube

In group A, 8F ultrafine chest drainage tubes (ABLE®; Baihe, Guangdong, China) were placed postoperatively. The upper tube was placed in the 2nd intercostal space of the midclavicular line, and the lower tube was placed in the 7th ~ 9th intercostal space of the posterior axillary line. In group B, 24F chest drainage tubes (Cobonyy®; Kebang, Suzhou, China) were placed along the 7th intercostal observation hole after surgery. See Fig. 1.

After operation, electrocardiogram (ECG) was performed, vital signs were monitored continuously, routine fluid was supplemented and drainage tube connected to a three-chamber drainage bottle to maintain negative pressure. Blood gas analysis, electrolyte analysis and bedside chest radiograph were reviewed on day one after operation. Computed tomography (CT) examination was performed on day 3 after operation. Nursing care of thoracic drainage was provided to ensure unobstruction of drainage tube.

The criteria for chest tube removal were as follows: (1) drainage volume of < 50 mL in 24 h; (2) absence of intrathoracic hemorrhage and air leakage, and (3) absence of signs of pleural effusion and atelectasis. The criteria for discharge were as follows: (1) Chest drainage tube should be removed; (2) Inflammatory parameters should be trending down and close to normal (white blood cell count < 12,000/mm ^3^); (3) Absence of fever (temperature ≤ 38 °C).

Postoperative pain was evaluated by visual analogue scale [12], wherein each patient subjectively scores a postoperative pain scores from 0 (no pain) to 10 (severe pain). Pain scores were recorded on postoperative days (POD) 1, 2 and 3. If the patient had obvious pain, 30–60 mg ketorolac was injected intramuscularly, every 4 to 6 h, according to the patients’ age and weight, and the medication should be discontinued as soon as the pain changes. The observation indicators included total postoperative drainage volume, drainage days, postoperative hospital stay, and postoperative complications (including subcutaneous emphysema, pulmonary infection, atelectasis, chest tube reinsertion, and intrathoracic hemorrhage).

### Statistical analysis

SPSS 26.0 software was adopted for PSM and data analysis (IBM, Armonk, NY). Multiple logistic regression analysis was used to evaluate propensity scores for all patients. Variables in the match include gender, age, tumor location, postoperative pathological staging and pathological types. The patients with the closest propensity values were matched at 1:1. Matching tolerance was set to 0.02. Continuous variables were expressed as means ± standard deviation (SD). Categorical variables were expressed as frequencies and percentages. Significant differences between the groups were assessed using student’s t test for continuous variables, and χ^2^ test for categorical variables. Mann-Whitney test was used for ordinal categorical variables. A *p*-value of less than 0.05 was considered as significance for all analyses.

## Results

There was no significant difference in the general data between the two groups, indicating no statistical significance (*P* > 0.05) (Table 1). Compared with Group B, Group A patients had significant advantages of pain scores (POD 1, 2 and 3), postoperative drainage volume, drainage days, and postoperative hospital stay (*P* < 0.05), (Table 2).

**Table 2:** Visual analogue scale scores, Drainage time, Total drainage volume and Postoperative hospital stay

Postoperative observation indicators	Group A (*n* = 67)	Group B (*n* = 67)	*P*-value
Visual analogue scale score			
POD 1	3.72 ± 0.65	3.94 ± 0.67	0.027
POD 2	2.72 ± 0.93	3.13 ± 1.04	0.016
POD 3	1.87 ± 0.65	2.39 ± 1.22	0.005
Drainage time (d)	4.25 ± 1.79	6.04 ± 1.96	0.000
Total drainage volume (ml)	1100.42 ± 701.57	1369.39 ± 624.25	0.021
Postoperative hospital stay (d)	8.46 ± 2.48	9.37 ± 1.70	0.014

Scale variables were expressed as median and range, and ordinal and nominal parameters as absolute numbers, and percent.

*POD* Postoperative days

Postoperative complications recorded in the two groups were subcutaneous emphysema, pulmonary infection, atelectasis, chest tube reinsertion, and intrathoracic hemorrhage, which showed no significant differences (*P* > 0.05), (Table 3). Thirty-seven cases had subcutaneous emphysema on 1 ~ 3 days after operation, and 33 cases of these had mild subcutaneous emphysema, which was cured by full exhaustion of drainage through thoracic drainage tube, and the remaining 4 cases were cured after chest tube reinsertion due to drainage tube dislocation or poor drainage. Ten cases had pulmonary infection on 3 ~ 4 days after operation, and were cured by ECG monitoring, anti-infection treatment and nutritional support. Eleven patients had atelectasis on 3 ~ 4 days after operation, and all of them showed poor performance of active cough and sputum excretion. Two cases had thoracic hemorrhage in the postoperative recovery room. The patients were cured and discharged from the hospital after undergoing thoracotomy in time, which showed effective hemostasis.

**Table 3:** Postoperative complications

Variable	Group A (*n* = 67)	Group B (*n* = 67)	*P*-value
Subcutaneous emphysema (%)	25.37%	29.85%	0.562
Pulmonary infection (%)	5.97%	8.96%	0.511
Atelectasis (%)	5.97%	10.45%	0.345
Chest tube reinsertion (%)	4.48%	1.49%	0.310
Intrathoracic hemorrhage (%)	2.99%	0%	0.154

Scale variables were expressed as median and range, and ordinal and nominal parameters as absolute numbers, and percent

## Discussion

The routine use of chest drainage tube after lobectomy aids pleural effusion that is discharged from the body, eliminate the residual cavity of the chest and promote the reexpansion of the lung. It is very important to reduce pulmonary infection and timely detect intrathoracic bleeding and other postoperative complications [5]. Therefore, when selecting the chest drainage tube, the safety and effectiveness of patients should be considered first [13]. With the promotion and use of the concept of ERAS, minimally invasive surgery has deeply rooted in the hearts of the people in recent years [10]. With this, we realized that postoperative pain and diaphragm stimulation caused by thick chest tubes might not be conducive in accelerating the recovery of patients after operation. So, it is of great clinical significance to explore whether an 8F ultrathin chest drainage tube is safe and reliable when compared with traditional thick chest drainage tube for accelerating the recovery of patients.

Due to the pressure of the drainage tube on the intercostal nerve and diaphragm, the placement of closed thoracic drainage tube can causes postoperative chest pain. This study showed statistically significant differences in pain scores between the two groups on POD 1, 2 and 3 after surgery. Pain scores of group A were significantly better than those in group B. Postoperative pain that affects the recovery of patients’ respiratory function and the risk of postoperative respiratory complications were increased. The postoperative pain was reduced, which in turn enhanced the initiation of cough and sputum, promoted lung expansion, reduced lung infection, and was more conducive to ambulation.

**Fig. 2:**
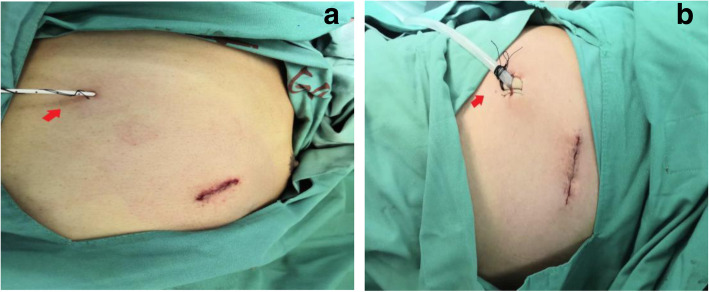
The appearance of placing chest drainage tube; **a** 8F chest drainage tube is placed; **b** 24F chest drainage tube is placed and reserved sutures can be seen at the drainage orifice

The insertion of an 8F ultrafine chest drainage tube is simple, and extubation remains to be more convenient and quick. After extubation, the incision was closed naturally and cannot be easily injected into air by just apply the normal dressing externally. However, in order to avoid the intake of air or leakage of drainage outlet after extubation of 24F chest drainage tube, vaseline gauze or reserved suture ligation is warranted, but it is more complicated and risky. This leaves a long surgical scar after healing, and affects the appearance, leaving a psychological trauma that is difficult to heal for the patient. After switching to an ultrafine chest drainage tube, the incision remained small, the perivascular tissue inflammatory response was shown to be mild, and the postoperative scar was small, making it more beautiful. See Fig. 2.

**Fig. 3:**
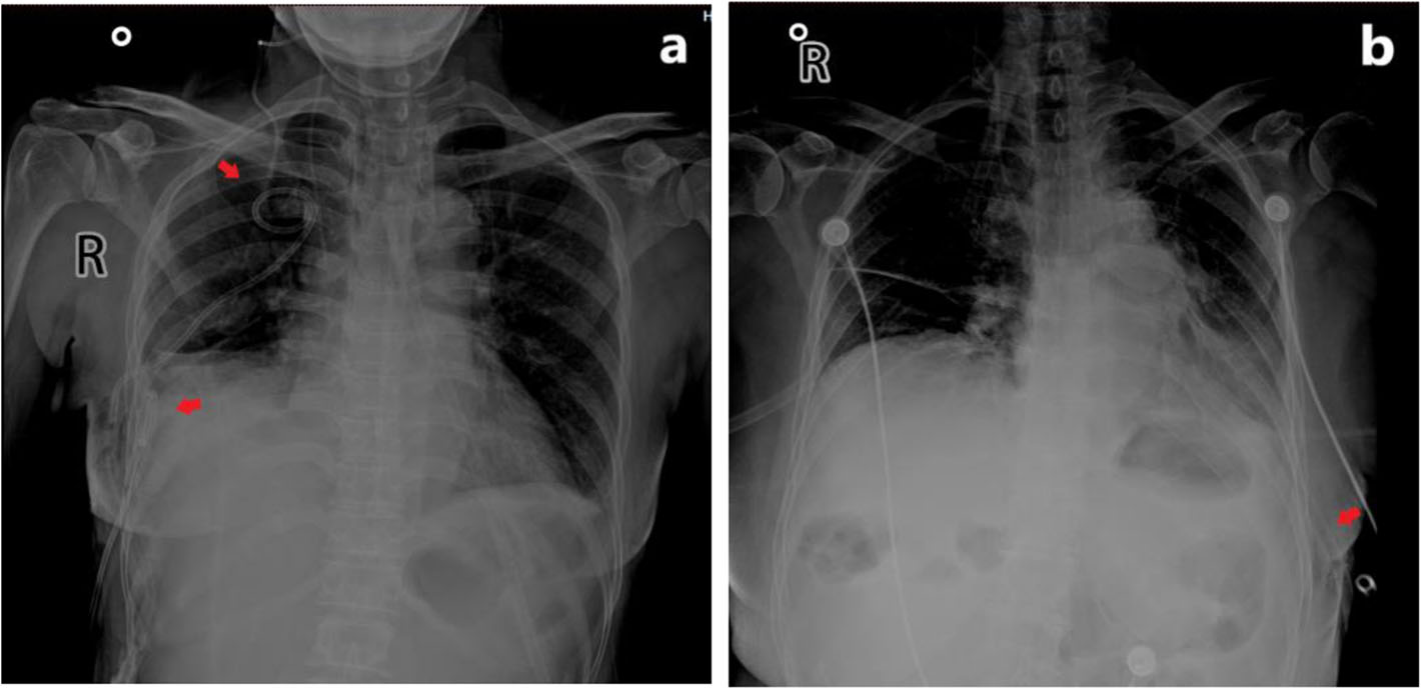
Postoperative X-ray of the thoracic cavity of patients; **a** the arrows indicate that two 8F chest drainages tubes are placed in the thoracic cavity; **b** the arrows indicate that one 24F chest drainage tube is placed in the thoracic cavity

In this study, the drainage days in group A were shorter than those in group B, and the postoperative hospital stay in group A was shorter than those in group B, and the total postoperative drainage volume was also lower than that in group B, showing statistically significant differences. The inner wall of an ultrafine chest drainage tube is smooth, with strong anti-coagulation ability and good flexibility. It can be coiled in the costophrenic angle or followed between the lung and chest wall, making the drainage tube more smooth and sufficient. However, due to thick texture of the 24F chest drainage tube, it is not easy to be completely placed in the costophrenic angle or followed between the chest wall and the lung lobe. Therefore, it might compress the lung lobe and diaphragm muscle, stimulating the production of pleural effusion, thus prolonging the drainage period. See Fig. 3.

Although the inner diameter of an 8F ultrafine chest drainage tube is smaller than that of traditional 24F drainage tube, patients ambulate earlier, promote fluid accumulation and faster drainage due to its advantage in pain management, and the risk of atelectasis and pulmonary infection does not increase significantly when compared with thick drainage tube. If the lung was well recovered and had cough without bubble overflow, the patients using an 8F ultrafine chest drainage tube can replace the water-sealed drainage bottle as the drainage bag, and so the patients can ambulate more easily, making it more convenient for thin drainage tube.

For patients with postoperative air leakage, high glucose can be injected into the chest to promote thoracic adhesion. The operation of an 8F ultrafine chest drainage tube is simple and aseptic, while drug injection into the thoracic cavity through traditional 24F chest drainage tube remains tedious and easily contaminated.

Among the 67 patients in group A, 2 patients had intrathoracic hemorrhage in the postoperative resuscitation room and so underwent secondary surgery for hemostasis, and all of them were cured and discharged. Although the 8F ultrafine chest drainage tube had a thicker and smaller inner diameter, intrathoracic hemorrhage in time and effectively could still be found.

An 8F ultrafine chest drainage tube is also associated with several problems: (1) among the cases in group A, the reason for chest tube reinsertion in 3 patients was drainage tube dislocation. Therefore, the depth of the catheter should be flexibly grasped according to the thickness of the chest wall in clinical practice. It should not be too shallow or too deep, in which too shallow might depart the drainage tube, and too deep might bend the drainage tube into an angle in the chest cavity that affects the drainage; and (2) the ultrafine chest drainage tube should be placed at another puncture point, and not through surgical incision. This is because if the tissue around the tube is not dense enough, then there might be fluid seepage around the mouth of the tube; and exudation of drainage orifice might also occur after extubation.

## Conclusion

In conclusion, the application of 8F ultrafine chest drainage tube after thoracoscopic lobrectomy can reduce postoperative pain, fully drain, facilitate ambulation, accelerate postoperative recovery, and do not increase the risk of postoperative complications such as subcutaneous emphysema, pulmonary infection, and atelectasis. At the same time, postoperative thoracic hemorrhage can be detected timely and effectively, leading to an effective, safe and reliable way of drainage.

## Data Availability

The datasets used and analysed during the current study are available from the corresponding author on reasonable request.

## Abbreviations

ERAS: Enhanced recovery after surgery
VATS: Video-assisted thoracoscopic surgery
MLND: Mediastinal lymph node dissection
CT: Computer tomography
POD: Postoperative days
SD: Standard deviation
